# Sleep behavior in relation to body mass index in primary school students

**DOI:** 10.3389/fpubh.2025.1703793

**Published:** 2025-12-11

**Authors:** Guiyun Wang, Yepeng Cai, Ye Zhang, Yanxia Qi, Lei Yang, Liu Yang, Yuexia Liao

**Affiliations:** 1 School of Nursing, Shandong Xiehe University, Jinan, China; 2 School of Nursing and Public Health, Yangzhou University, Yangzhou, China

**Keywords:** primary school students, sleep duration, bedtime, social jet lag, insufficient sleep, overweight and obesity

## Abstract

**Objective:**

This study aims to examine the relationship between multiple sleep behaviors in elementary school students—including occasional late bedtimes, social jet lag, and weekend oversleeping—and subsequent changes in their body mass index over 1 year.

**Methods:**

A one-year cohort study was carried out in Yangzhou City, Jiangsu Province, China, involving 3,213 students in grades one through five. At baseline and follow-up, trained staff obtained height and weight through standardized on-site measurements, and body mass index (BMI) was calculated. According to changes in BMI, students were assigned to four categories. Different sleep behaviors were collected through self-reported questionnaires. Multiple logistic regression was applied to assess the association between these sleep behaviors and BMI change.

**Results:**

The normal weight persistence group (C1), overweight obesity reduction group (C2), overweight obesity increase group (C3), and overweight obesity persistence group (C4) consisted of 1,508 (46.93%), 678 (21.10%), 191 (5.94%), and 836 (26.02%) children, respectively. Social jet lag varied across different groups of lower-grade children. The monthly maximum weekend bedtime—defined as the latest time a child went to bed on any weekend night within that month—was identified as a contributing factor to overweight and obesity. Weekday and weekend sleep opportunities influenced overweight and obesity among upper-grade students.

**Conclusion:**

The monthly maximum weekend bedtime is an important indicator related to the development and continuation of overweight and obesity in elementary school students. Sleeping opportunity assessments should clearly separate school days from weekends.

## 1 Introduction

Overweight and obesity have emerged as major global public health challenges, affecting nearly one-third of the population across all ages. Over the past three decades, childhood obesity rates have risen by 2.44 times (1, 2). By 2030, more than 100 million children aged 5–9 and over 150 million adolescents aged 10–19 are expected to be affected (3). The impact of childhood overweight obesity often extends into adulthood without timely intervention (4, 5). Previous studies have shown that obesity is strongly associated with diabetes, heart disease, asthma, arthritis, and depression (6). Identifying modifiable factors that may prevent or reduce childhood overweight obesity is therefore essential.

With rapid socio-economic growth and lifestyle changes, sleep problems among children and adolescents have become increasingly noticeable and are now a major concern in the field of public health. Common sleep issues include irregular sleep patterns, low sleep satisfaction, circadian rhythm disturbances, poor sleep efficiency, and inadequate or excessive sleep duration (7). Sleep deprivation is widespread, with over one-third of children in the Americas, 42.1% in Europe, and 57.15% in Asia sleeping less than recommended (8–10). Poor sleep habits in childhood have been linked to inflammation (11), increased risk of myopia (12), negative neurodevelopmental outcomes (13), and obesity (14). In addition, elementary school students attend school 5 days per week with two rest days, which can disrupt circadian rhythm more frequently than severe misalignment (15). This disruption is associated with reduced 24-h energy expenditure and altered appetite hormone levels, thereby increasing obesity risk (16, 17).

Primary school is a crucial period for growth and metabolic regulation, during which inadequate sleep behaviors may shape long-term weight patterns (18). Although previous research has reported associations between sleep loss and elevated BMI in children (19), key gaps remain. Most available studies examine single sleep indicators, such as total duration, rather than the combined influence of multiple behaviors—such as sporadic late bedtimes, social jet lag, and weekend oversleep—that frequently occur together in daily life (20). Furthermore, longitudinal evidence on how combined sleep behaviors affect weight change among primary school students is limited, particularly in China, which has the world’s largest population of children with obesity (21). Understanding the long-term effects of irregular sleep patterns requires evaluating multiple sleep dimensions rather than relying on isolated indicators.

The aim of this study was to analyze how various sleep behaviors, including occasional late bedtimes, social jet lag, and weekend oversleep, are associated with one-year changes in weight status among primary school students.

## 2 Materials and methods

### 2.1 Materials source

**Figure 1:**
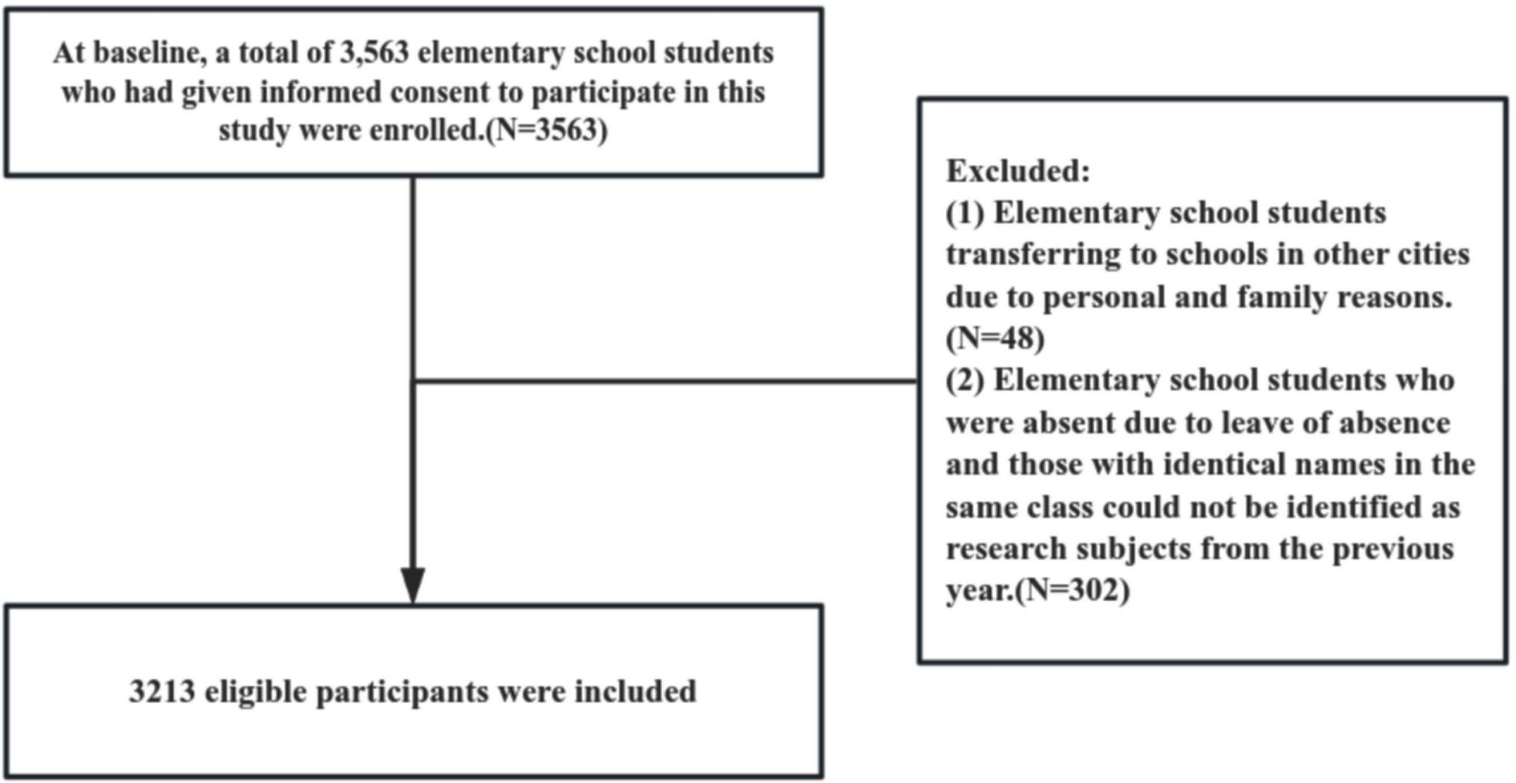
Flow chart of study participants.

In November 2021, we recruited all primary school students in grades 1–5 from seven urban schools in Yangzhou City, Jiangsu Province, a region with relatively advanced economic development, using whole-cluster sampling. One year later, participants completed a second round of height and weight measurements, and all data were matched by name, class, and school (Figure 1).

### 2.2 Inclusion and exclusion criteria

The inclusion criteria were: (1) Students enrolled in grades 1–5; (2) Children with local household registration as the main target population. Children with non-local registration, those who had recently moved to the city, or temporary residents whose residence duration exceeded two-thirds of their age were also eligible. Exclusion criteria included: (1) Children with communication difficulties or severe physical illness; (2) Participation in other research projects. A total of 3,563 students provided informed consent. Among them, 3,213 completed follow-up, while 350 were lost, resulting in a 9.83% loss-to-follow-up rate.

### 2.3 Ethical approval

This study complied with the Declaration of Helsinki and followed the STROBE reporting guidelines. Ethical approval was granted by the Ethics Committee of the School of Nursing–College of Public Health, Yangzhou University (Approval number: YZUHL20210103). Parents or guardians signed consent forms, and students provided assent before participation.

### 2.4 Covariate assessment

In the questionnaire, participants provided demographic information, including gender (male/female), grade level (grades 1–5), ethnicity (Han Chinese/ethnic minority), and only-child status (yes/no).

### 2.5 Exposure assessment

Total daily sleep duration was obtained through self-reported items, including: “On weekdays, what time do you usually wake up? What time do you take naps? What is your average bedtime? What was your earliest bedtime in the past month? What was your latest bedtime in the past month?” and similar questions for weekends. Sleep opportunity was used to estimate total sleep duration (22), calculated as the sum of nighttime sleep opportunity and nap duration. Social jet lag (23) was defined as the absolute difference between the midpoint of sleep on non-school days and the midpoint of sleep on school days. Oversleep on non-school days (22) was defined as the difference in sleep opportunity between non-school days and school days. Explanation of sleep variables and calculation methods are presented in Table 1.

**Table 1:** Sleep variable calculation method.

Sleep variables	Calculation method
Sleep opportunity^*^	night time sleep + nap
Bedtime	When bedtime is before midnight: bedtime = Original value When bedtime falls in the early hours of the next day = Original value+24
Earliest bedtime/Latest bedtime	The earliest or latest bedtime within a month
Social Jetlag	Midpoint of sleep duration on weekend – Midpoint of sleep duration on weekdays
Weekend oversleeping	weekend sleep opportunity – weekday sleep opportunity

*When bedtime is before midnight: nighttime = (24 – bedtime) + wake time, When bedtime falls in the early hours of the next day: nighttime = wake time – early morning hours; The midpoint of sleep is the halfway point between falling asleep and waking up; for example, if bedtime is 12:00 a.m. and wake-up time is 8:00 a.m., the midpoint of sleep is 4:00 a.m.

### 2.6 Outcome assessment

Body weight change over a one-year follow-up period served as the study outcome. Body Mass Index (BMI) was calculated from measured height and weight at baseline and at 1 year, and participants were classified into four groups.

#### 2.6.1 Outcome metric definition

Participants were categorized into four groups based on changes in body weight over the one-year period: consistently normal body weight (C1), reduced overweight and obesity (C2), increased overweight and obesity (C3), and persistent overweight and obesity (C4).

1. Consistently normal body weight (C1): Weight was normal at baseline and remained normal at one-year follow-up.
2. Reduced overweight and obesity (C2): Baseline weight was overweight or obese but returned to normal after 1 year.
3. Increased overweight and obesity (C3): Baseline weight was normal, but increased to overweight or obese after 1 year.
4. Persistent overweight and obesity (C4): Baseline weight was overweight or obese and remained so at the one-year follow-up.

#### 2.6.2 Measurement method

At both baseline and follow-up, height and weight were measured on-site to calculate BMI, with identification based on school, class, and full name. BMI classification followed the “Screening for Overweight and Obesity in School-Aged Children and Adolescents” standard issued by the National Health and Wellness Commission of China (24).

Overweight: BMI ≥ the 85th percentile and < the 95th percentile of reference values for children and adolescents of the same age and gender.

Obesity: BMI ≥ the 95th percentile of reference values for children and adolescents of the same age and gender.

To ensure accuracy and consistency, pediatricians and medical staff underwent standardized training, and all measuring instruments were regularly calibrated. Height was measured using the HXBM-19 Martin Altimeter, and weight was measured using the SECA-877 medical electronic scale, following the Technical Code for Physical Examination of Students (GB/T26343-2010) (25).

### 2.7 Statistical analysis

The data were entered into Epidata 3.0 and subsequently verified. A cohort of elementary school students was established using school, class, and name controls to determine the height and weight of each primary school student after 1 year. The elementary school students were then categorized into the following groups: The participants were classified into four groups based on their one-year change in body weight: consistently normal body weight (C1), reduced overweight and obesity (C2), increased overweight and obesity (C3), and persistent overweight and obesity (C4). The data were analyzed using SPSS 26.0 software. The normality test was used to assess the distribution of the measurement data. Data that conformed to a normal distribution were expressed as mean ± standard deviation, while data that did not conform to a normal distribution were expressed as median (interquartile range). The data on count variables were expressed as frequency and percentage. To compare the differences between the groups, either an independent samples *t*-test or one-way ANOVA was used, with a difference considered statistically significant at *p* < 0.05. The correlation was analyzed by multinomial logistic regression analyses.

## 3 Results

### 3.1 Collection on study participants

A total of 3,563 primary school students consented to participate at baseline, 3,213 completed the one-year follow-up, and 350 were lost to follow-up, yielding a loss rate of 9.83%. The numbers in groups C1, C2, C3, and C4 were 1,508 (46.93%), 678 (21.10%), 191 (5.94%), and 836 (26.02%), respectively.

### 3.2 Baseline characteristics of participants

There were 1,665 males and 1,548 females. The proportion of females was higher in groups C1 and C3, whereas males were more represented in groups C2 and C4, with statistically significant differences. The proportion of students in the C1 group decreased with increasing grade level. Fifth-grade students had the highest rate of overweight and obesity reduction, while lower-grade students showed a higher rate of overweight and obesity after 1 year. Upper-grade students had higher rates of persistent overweight and obesity, with differences reaching statistical significance (*p* < 0.001). Ethnicity and only-child status did not differ significantly among groups (Table 2).

**Table 2:** General information questionnaire for primary school students (*n* = 3,213).

Characteristics	C1	C2	C3	C4	*X^2^*	*P*
Gender						
Males	622 (37.38)	397 (23.84)	54 (3.24)	592 (35.56)	243.055	<0.001
Females	886 (57.24)	281 (18.15)	137 (8.85)	244 (15.76)		
Nation						
Han	1,475 (46.93)	663 (21.09)	187 (5.95)	818 (26.03)	0.013	1.000
Other	33 (47.14)	15 (21.43)	4 (5.71)	18 (25.71)		
Grade						
Grade 1	146 (60.08)	43 (17.70)	19 (7.82)	35 (14.40)	80.074	<0.001
Grade 2	227 (55.10)	82 (19.90)	27 (6.55)	76 (18.45)		
Grade 3	220 (52.63)	70 (16.75)	23 (5.50)	105 (25.12)		
Grade 4	518 (46.00)	220 (19.54)	62 (5.51)	326 (28.95)		
Grade 5	397 (39.15)	263 (25.94)	60 (5.92)	294 (28.99)		
Only child						
Yes	780 (45.99)	361 (21.29)	93 (5.48)	462 (27.24)	4.083	0.253
No	728 (47.99)	317 (20.90)	98 (6.46)	374 (24.65)		

### 3.3 Sleep status of primary school children with different BMI classification changes

Survey findings show that among lower-grade students, Group C1 reported the earliest bedtime on school days, while Group C3 reported the latest. Group C2 had the earliest average weekend bedtime, whereas Groups C3 and C4 tended to go to bed later. Groups C1 and C2 also reported significantly earlier weekend bedtimes than Groups C3 and C4. Social jet lag among lower-grade students followed this pattern: C2 < C1 < C4 < C3 (Table 3). Among upper-grade students, Groups C1 and C2 showed significantly earlier school-day bedtimes compared with Groups C3 and C4. Group C1 also had earlier monthly maximum weekend bedtimes than the other three groups, and its weekend sleep opportunity was higher than in Groups C2, C3, and C4 (Table 4). Furthermore, in grades 1 through 5, the earliest and latest monthly bedtimes differed by more than 1 h.

**Table 3:** Sleep status of primary school students with different BMI classification changes in grades 1–3 (*n* = 1,073).

Sleep behavior	C1	C2	C3	C4	*F*	*P*
School Wake-Up Time	7:00 ± 0.24	6:59 ± 0.25	6:58 ± 0.26	6:56 ± 0.27	3.083	0.027
School nap time	12.10 ± 20.35	12.10 ± 19.65	12.97 ± 20.94	14.56 ± 22.62	0.796	0.496
School bedtime	21:20 ± 0.48	21:22 ± 0.47	21:19 ± 0.52	21:21 ± 0.50	0.318	0.812
School earliest bedtime	20:53 ± 0.55	20:54 ± 0.53	20:52 ± 0.63	20:51 ± 0.57	0.343	0.794
School latest bedtime	21:59 ± 0.59	22:08 ± 0.65	22:12 ± 0.75	22:07 ± 0.67	4.057	0.007
Weekend Wake-Up Time	7:56 ± 0.67	7:48 ± 0.68	7:57 ± 0.79	7:53 ± 0.80	1.802	0.145
Weekend nap time	18.33 ± 29.74	23.03 ± 30.77	19.06 ± 29.07	21.14 ± 33.56	1.320	0.267
Weekend bedtime	21:38 ± 0.55	21:32 ± 0.54	21:47 ± 0.69	21:47 ± 0.71	7.412	<0.001
Weekend earliest bedtime	21:08 ± 0.61	21:05 ± 0.62	21:13 ± 0.72	21:11 ± 0.76	1.282	0.279
Weekend latest bedtime	22:12 ± 0.64	22:07 ± 0.83	22:25 ± 0.75	22:26 ± 0.87	20.059	<0.001
School sleeping opportunity	9.66 ± 0.49	9.61 ± 0.49	9.66 ± 0.53	9.59 ± 0.55	1.365	0.252
Weekend sleeping opportunity	10.31 ± 0.74	10.20 ± 0.68	10.17 ± 0.90	10.14 ± 0.95	3.09	0.026
Social Jetlag	0.64 ± 0.42	0.50 + 0.41	0.73 ± 0.55	0.70 ± 0.59	8.199	<0.001
Weekend oversleeping	0.76 ± 0.65	0.72 ± 0.57	0.69 ± 0.68	0.81 ± 0.74	0.832	0.476

**Table 4:** Sleep status of primary school students with different BMI classification changes in grades 4-5 (*n* = 2,140).

Sleep behavior	C1	C2	C3	C4	*F*	*P*
School Wake-Up Time	6:45 ± 0.42	6:43 ± 0.43	6:43 ± 0.45	6:41 ± 0.48	2.022	0.109
School nap time	15.75 ± 27.04	14.82 ± 28.61	17.34 ± 26.97	16.49 ± 29.15	0.446	0.720
School bedtime	21:07 ± 0.65	21:02 ± 0.65	21:10 ± 0.79	21:15 ± 0.86	9.299	<0.001
School earliest bedtime	20:26 ± 0.90	20:20 ± 0.79	20:21 ± 1.077	20:22 ± 0.88	1.487	0.216
School latest bedtime	22:08 ± 1.04	22:06 ± 1.06	22:18 ± 1.12	22:12 ± 1.22	1.556	0.198
Weekend Wake-Up Time	7:49 ± 1.13	7:45 ± 1.14	7:35 ± 1.11	7:43 ± 1.19	1.786	0.148
Weekend nap time	29.17 ± 38.08	30.06 ± 40.75	29.34 ± 34.92	31.48 ± 39.48	0.453	0.715
Weekend bedtime	21:16 ± 0.91	21:19 ± 0.95	21:26 ± 0.87	21:22 ± 0.92	2.507	0.057
Weekend earliest bedtime	20:30 ± 0.93	20:35 ± 0.97	20:35 ± 0.95	20:34 ± 0.94	0.999	0.392
Weekend latest bedtime	22:13 ± 1.09	22:24 ± 1.32	22:29 ± 1.16	22:27 ± 1.26	5.836	0.001
School sleeping opportunity	9.63 ± 0.76	9.69 ± 0.76	9.56 ± 0.83	9.57 ± 0.81	2.461	0.061
Weekend sleeping opportunity	10.55 ± 1.31	10.45 ± 1.26	10.15 ± 1.39	10.34 ± 1.36	5.119	0.002
Social Jetlag	0.71 ± 0.63	0.77 ± 0.66	0.70 ± 0.54	0.75 ± 0.62	1.035	0.376
Weekend oversleeping	1.13 ± 1.03	1.06 ± 1.00	1.12 ± 1.11	1.13 ± 1.09	0.636	0.592

### 3.4 Analysis of the association between changes in different BMI classifications and sleep behavior

Elementary school students divided into four BMI categories served as the dependent variable. Variables with *p* < 0.1 in univariate analysis were included as independent variables in the multinomial logistic regression model. The scoring approach is presented in Table 5. Results indicate that among lower-grade students, girls showed a higher likelihood of becoming overweight or obese after 1 year than boys. A later monthly maximum weekend bedtime was a risk factor for increased overweight and obesity. Among female students, the likelihood of persistent overweight or obesity was 0.263 times that of male students, and this risk increased by 1.336 times for each advancing grade level (Table 6). Reduced weekend sleep opportunity may also contribute to overweight and obesity among fourth- and fifth-grade students. Among upper-grade students, those in the C4 group were more likely to have later school-day and weekend bedtimes, along with fewer sleep opportunities on both school days and weekends. These patterns were less common in the group (C1) (Table 7). A later monthly maximum weekend bedtime remained a risk factor for overweight and obesity across all grade levels (Tables 6, 7).

**Table 5:** Variables and assignment method.

Variant	Assignment method
Implicit variable	
Changes in BMI classification of primary school children	C1 group = 0; C2 group = 1; C3 group = 2; C4 group = 3
Independent variable	
Genders	Male = 1; Female = 2
Grade	Original value input
School Wake-Up Time	Original value input
School latest bedtime	Original value input
Weekend Bedtime	Original value input
Weekend latest bedtime	Original value input
Weekend sleeping opportunity	Original value input
Social Jetlag	Original value input
School bedtime	Original value input
Weekend bedtime	Original value input
Weekend latest bedtime	Original value input
School sleeping opportunity	Original value input
Weekend sleeping opportunity	Original value input

**Table 6:** Analysis of the association between weight change and sleep behaviors in grades 1–3 (*n* = 1,073).

Group	Sleep behavior	*β*	Sx	Wald *χ*^2^	*P*	OR (95%CI)
C2	Genders	−1.955	0.206	89.731	<0.001	0.142 (0.096–0.217)
	Grade	0.082	0.118	0.483	0.487	1.085 (0.860–1.365)
	School wake-up time	−0.618	0.417	2.202	0.138	0.539 (0.368–1.153)
	School latest bedtime	0.237	0.186	1.625	0.202	1.267 (0.733–1.796)
	Weekend Bedtime	0.146	0.353	0.172	0.679	1.157 (0.435–1.599)
	Weekend latest bedtime	−0.075	0.193	0.151	0.698	0.928 (0.485–1.476)
	Weekend sleeping opportunity	0.121	0.221	0.299	0.584	1.128 (0.630–1.731)
	Social Jetlag	−0.813	0.401	4.108	0.043	0.443 (0.135–0.973)
C3	Genders	1.262	0.365	11.983	0.001	3.532 (1.728–7.180)
	Grade	−0.271	0.173	2.438	0.118	0.763 (0.553–1.095)
	School Wake-Up Time	−0.209	0.625	0.111	0.738	0.812 (0.477–1.368)
	School latest bedtime	−0.124	0.239	0.271	0.602	0.883 (0.593–1.345)
	Weekend Bedtime	−0.83	0.444	3.498	0.061	0.436 (0.136–1.068)
	Weekend latest bedtime	1.206	0.236	26.194	<0.001	3.341 (2.043–5.114)
	Weekend sleeping opportunity	−0.407	0.283	2.074	0.150	0.666 (0.453–1.115)
	Social Jetlag	0.285	0.486	0.346	0.557	1.330 (0.472–2.773)
C4	Genders	−1.334	0.176	57.573	<0.001	0.263 (0.186–0.371)
	Grade	0.290	0.115	6.392	0.011	1.336 (1.073–1.683)
	School Wake-Up Time	−1.029	0.373	7.589	0.006	0.357 (0.228–0.630)
	School latest bedtime	0.111	0.166	0.448	0.503	1.118 (0.672–1.493)
	Weekend Bedtime	0.094	0.297	0.100	0.752	1.098 (0.475–1.773)
	Weekend latest bedtime	0.391	0.172	5.160	0.023	1.479 (1.032–2.019)
	Weekend sleeping opportunity	−0.093	0.182	0.263	0.608	0.911 (0.583–1.335)
	Social Jetlag	0.033	0.332	0.010	0.920	1.034 (0.332–3.053)

**Table 7:** Analysis of the association between weight change and sleep behaviors in grades 4–5 (*n* = 2,140).

Group	Sleep behavior	*β*	Sx	Wald *χ*^2^	*P*	OR (95%CI)
C2	Genders	−0.200	0.115	3.051	0.081	0.818 (0.654–1.025)
	Grade	0.410	0.114	12.817	<0.001	1.507 (1.204–1.885)
	School bedtime	−0.277	0.152	3.316	0.069	0.758 (0.563–1.021)
	Weekend bedtime	0.069	0.097	0.503	0.478	1.071 (0.886–1.296)
	Weekend latest bedtime	0.070	0.063	1.229	0.268	1.072 (0.948–1.212)
	School sleeping opportunity	−0.004	0.132	0.001	0.974	0.996 (0.769–1.290)
	Weekend sleeping opportunity	−0.039	0.052	0.547	0.460	0.962 (0.868–1.066)
C3	Genders	0.348	0.203	2.95	0.086	1.416 (0.952–2.107)
	Grade	0.178	0.194	0.837	0.360	1.195 (0.816–1.749)
	School bedtime	0.027	0.255	0.011	0.915	1.028 (0.624–1.693)
	Weekend bedtime	−0.074	0.165	0.198	0.656	0.929 (0.672–1.285)
	Weekend latest bedtime	0.150	0.100	2.255	0.133	1.161 (0.955–1.411)
	School sleeping opportunity	0.100	0.224	0.199	0.656	1.105 (0.712–1.715)
	Weekend sleeping opportunity	−0.233	0.091	6.521	0.011	0.792 (0.662–0.947)
C4	Genders	−1.168	0.113	106.838	<0.001	0.311 (0.249–0.388)
	Grade	0.140	0.109	1.629	0.202	1.150 (0.928–1.425)
	School bedtime	0.550	0.14	15.513	<0.001	1.734 (1.318–2.280)
	Weekend bedtime	−0.165	0.09	3.323	0.068	0.848 (0.710–1.012)
	Weekend latest bedtime	0.146	0.058	6.394	0.011	1.158 (1.033–1.297)
	School sleeping opportunity	0.315	0.127	6.200	0.013	1.371 (1.069–1.757)
	Weekend sleeping opportunity	−0.116	0.049	5.535	0.019	0.891 (0.809–0.981)

## 4 Discussion

This study examined the demographic characteristics of participants with shifts in BMI classification and their associations with sleep behaviors (bedtime, sleep opportunity, social jet lag, and occasional late nights) among 3,563 elementary school students in East China. Of these, 3,213 completed the one-year follow-up (loss rate 9.83%). Key findings indicate that the monthly maximum weekend bedtime is closely related to overweight and obesity in elementary school students. Sleep opportunities on weekdays and weekends also showed significant associations with overweight and obesity among upper-grade students. Social jet lag differed among lower-grade students across BMI groups.

This study found that the later the monthly maximum weekend bedtime, the greater the risk of persistent overweight and obesity. Among younger students, a later monthly maximum weekend bedtime was linked to becoming overweight or obese. Occasional late nights on weekends, although not chronic sleep disorders, can disrupt circadian secretion patterns, influencing appetite regulation and energy metabolism (15). Late-night activities often involve screen use or entertainment, accompanied by increased consumption of snacks or sugary drinks, leading to excess daily caloric intake (26, 27). Irregular sleep can also prolong sleep inertia and increase daytime fatigue, lowering outdoor activity and exercise levels and ultimately reducing energy expenditure (28, 29). This study innovatively assessed earliest and latest monthly bedtimes, a variable rarely discussed in existing literature. Occasional deviations from regular bedtime routines may disrupt circadian rhythms, and such disturbances may persist over several months.

Additionally, reduced weekend sleep opportunity may be associated with the development or maintenance of overweight and obesity, while greater sleep opportunity during school days may also contribute to weight gain. Previous studies have reported links between sleep loss and weight gain (15, 30). Increased school-day sleep opportunity may indicate high academic pressure, leading children to relieve stress with high-calorie snacks after class. Over time, these excess calories can accumulate and contribute to obesity (31). Excessive school-day sleep opportunity may also reflect reduced physical activity at school, lowering energy expenditure (32). These associations were more pronounced in upper-grade students, possibly because academic demands increase with grade level, reducing sleep time through homework and extracurricular classes (33). Irregular sleep habits may further affect sleep quality, complicating weight management (34).

This study also found that among younger students, social jet lag in Group C1 was significantly lower than in Groups C3 and C4, with Group C2 showing the smallest social jet lag. This is consistent with earlier studies reporting a positive association between social jet lag and weight changes in children. Social jet lag may disrupt appetite-regulating hormones, increasing appetite and caloric intake and promoting weight gain (35). The potential mechanism may involve circadian regulation of metabolism, sleep–wake cycles, feeding behavior, and hormone secretion (36). Students with greater social jet lag also tended to have later bedtimes, making them more likely to skip breakfast and snack after dinner. This pattern interacts with circadian misalignment and may contribute to overweight and obesity (37).

This study has several limitations. The survey relied on subjective reports rather than objective tools such as polysomnography or activity monitors. The monthly maximum bedtime can indicate the presence of late nights but not their frequency. Dietary data and chrononutrition-related variables were not collected, and genetic factors were not examined. Socio-economic information was limited and therefore omitted. These factors may introduce some bias into the findings.

## 5 Conclusion

In conclusion, the monthly maximum weekend bedtime is an important factor contributing to the development and persistence of childhood overweight and obesity. The impact of sleep opportunity on weight outcomes requires separate consideration for weekends and weekdays. Future research may incorporate objective sleep-monitoring tools to collect multidimensional data; include indicators reflecting the frequency of occasional late bedtimes; and combine chrononutrition variables such as dietary patterns, meal timing, and eating frequency to better understand how sleep–rhythm disruptions influence childhood weight.

## Data Availability

The datasets presented in this article are not readily available because based on the consent form indicating that the data will not be shared publicly for confidentiality, we will not share our data with the public. The datasets generated during the current study are not publicly available. Requests to access the datasets should be directed to YC, caiyepeng0312@163.com.
